# Conceptualizing the Commercialization of Human Milk: A Concept Analysis

**DOI:** 10.1177/08903344241254345

**Published:** 2024-06-10

**Authors:** Heather Christine Rusi, Laurence Grummer-Strawn, Maryanne Tigchelaar Perrin, Tracie Risling, Meredith Lee Brockway

**Affiliations:** 1 Faculty of Nursing, University of Calgary, Calgary, AB, Canada; 2 Alberta Children’s Hospital Research Institute, Calgary, AB, Canada; 3 Department of Nutrition and Food Safety, World Health Organization, Geneva, Switzerland; 4 Department of Nutrition, University of North Carolina Greensboro, Greensboro, NC, USA

**Keywords:** breastfeeding, commercialization, concept analysis, donor milk, health policy, human milk, infant nutrition, lactation, neonatology, pediatrics, regulation

## Abstract

**Background:**

Donor human milk is recommended when infants are unable to be fed their mother’s own milk or require supplementation. For-profit companies use technologies to create human milk products for infants in the neonatal intensive care setting without consistent guidelines and regulatory frameworks in place. This commercialization of human milk is inadequately conceptualized and ill-defined.

**Research Aims:**

The aim of this study is to conceptualize and define the commercialization of human milk and discuss the need for policy guidelines and regulations.

**Method:**

Using a concept analysis framework, we reviewed the literature on the commercialization of human milk, analyzed the antecedents and potential consequences of the industry, and developed a conceptual definition. The literature review resulted in 13 relevant articles.

**Results:**

There has been a surge in the development and availability of human milk products for vulnerable infants developed by for-profit companies. Commercialized human milk can be defined as the packaging and sale of human milk and human milk components for financial gain. Factors contributing to the commercialization of human milk include an increased demand for human milk, and consequences include potential undermining of breastfeeding. The lack of guidelines and regulations raises concerns of equity, ethics, and safety.

**Conclusion:**

The industry is rapidly growing, resulting in an urgent need for consistent guidelines and regulatory frameworks. If left unaddressed, there could be potential risks for donor milk banking, the future of breastfeeding, and infant and maternal health.

**Key Messages**

- There has been a surge in for-profit companies developing and selling human milk products targeted at vulnerable infants.
- Guidelines and regulations concerning human milk products for infants are inconsistent, leading to concerns of equity, ethics, and safety.
- Commercialized human milk products can be defined as the packaging and sale of human milk and human milk components for the purposes of financial gain.
- There is a need for strong policies to ensure ethics, safety, and the protection of vulnerable infants.

## Background

The benefits of breastfeeding and human milk are well-known globally. Breastfeeding and human milk feeding are one of the most effective ways to promote healthy growth and optimal development in early childhood (United Nations Children’s Fund [UNICEF], 2018; Victora et al., 2016). Breastfeeding can be defined as the child receiving human milk, either directly from the breast or expressed (World Health Organization [WHO], 1991). While the WHO (2001) recommends that infants exclusively breastfeed for the first 6 months of life, the rate of exclusive breastfeeding globally for infants under 6 months of age is only 48% (WHO & UNICEF, 2022). However, approximately 40% of infants in the neonatal intensive care (NICU) setting lack access to their mother’s own milk (MOM; Israel-Ballard, 2018). When infants are unable to be fed MOM or require supplementation, donor human milk (DHM) is the recommended alternative, particularly for those who are vulnerable (WHO & UNICEF, 2018). Evidence shows that DHM for preterm and sick infants in NICU settings is beneficial (WHO, 2011). Preterm infants fed with DHM compared to formula had lower rates of necrotizing enterocolitis (NEC; Quigley et al., 2019).

Historically, wet nursing was widely practiced to feed infants who were not breastfed by their own mothers (Jones, 2003). Wet nursing declined in the 20th century as technological innovation introduced artificial infant formula and modern bottle feeding (Stevens et al., 2009). Formula supplementation then became the substitute for breastfeeding. Increased availability of infant formula and marketing strategies of formula companies may have contributed to high supplementation rates (Rollins et al., 2023; Stevens et al., 2009).

Nonprofit milk banks were established in the early 1900s to provide DHM to vulnerable infants (Jones, 2003). Today, nonprofit milk banks receive DHM, which is processed and provided to infants, usually in the NICU setting (Human Milk Banking Association of North America [HMBANA], 2023). Many nonprofit milk banks are operated through milk banking associations which ensure processing and safety standards.

Since the early 2000s, human milk for infants has turned into a tradable commodity (Newman & Nahman, 2022). An increasing number of private enterprises that can distribute profits to their owners (in contrast to nonprofit organizations that do not distribute earned profits to their owners or members, but rather use the earned funds for the organization’s objectives; Kenton, 2023) utilize technologies to develop human milk products from expressed human milk (Hassan, 2010) or donated mammary cells (Cohen & Cassidy, 2022) to be sold at a profit (Newman & Nahman, 2022; Shenker et al., 2024; Smith, 2015). Human milk products were initially offered as multicomponent nutritional supplements, developed to increase nutrients for premature infants or infants with specific nutrient needs. Human milk-based fortifiers (HMFs) were developed as an alternative to bovine-based milk fortifiers for fortifying donor human milk. A 2017 survey found that 44% of advanced neonatal care hospitals in the United States were using a human milk-based fortifier developed by the world’s largest human milk company, Prolacta BioScience (Prolacta; Parker et al., 2020).

Historically, the commercial market for human milk has existed as food and medicine for adults and children (Cohen, 2019), however, over the past 20 years, there has been a surge in the number of for-profit human milk companies developing, marketing, and selling human milk products. Prolacta began in 1999 (Prolacta BioScience, 2022a) and the human milk industry has since expanded to include other companies globally, including Medolac Laboratories, NeoLacta Lifesciences (NeoLacta), and Biomilq. The history of infant feeding and the commercialization of human milk can be found in the online Supplemental Materials.

Prolacta claims that it has served more than 90,000 premature infants globally (Prolacta BioScience, 2022a) while NeoLacta has provided their human milk products to over 30,000 preterm babies (NeoLacta, 2022). The number and range of human milk products offered on the market is increasing, primarily targeted towards infants in NICU settings (see Table 1; Shenker et al., 2024). Currently, in most countries, these products are limited to the NICU settings where their use is supervised by a clinician. However, there is now an emergence of these products in the community where caregivers of infants who are not hospitalized can purchase products online, therefore expanding to the well-baby and full-term populations.

**Table 1.** A Snapshot of Commercialized Human Milk Products on the Market and in Development.

Product	Product Descriptions
Cell-cultured human milk	Laboratory-produced human milk Developed from mammary epithelial cells
Human donor milk (HDM)	HDM products include: pasteurized, frozen human milk. commercially sterile human milk stored at room temperature.
Human milk caloric fortifier or human milk cream	Pasteurized human milk cream with increased caloric content Intended for use with human milk and for improving the caloric density of human milk for preterm infants
Human milk fortifier (liquid and powder form)	Pasteurized, human-milk based product administered in addition to human milk Designed to meet specific nutritional needs of premature and low birth-weight infants
Human milk powder	100% human milk powder Ready to be reconstituted with water
Ready-to-feed human milk-based infant formula	Human milk-based, pasteurized, fortified donor milk product Mixture of human milk and human milk fortifier for premature infants

*Note*. The above products and product descriptions were taken from the following company websites: BIOMILQ, 2022; NeoKare, 2023; NeoLacta Lifesciences, 2022; Prolacta BioScience, 2022b.

While human milk products may be beneficial in some settings and are likely superior to infant formula in some contexts, current evidence demonstrates that processed human milk is not equivalent to human milk from the breast (Ames et al., 2023). Processed human milk lacks the dynamic nature and personalized nutrition that human milk from breastfeeding offers and is limited in many naturally occurring bioactive components and immune factors that contribute to infant growth, development, and immune function (Ames et al., 2023).

Currently, the commercialization of human milk, as a concept, is ambiguous and inadequately defined for healthcare providers and decision makers. The rise in for-profit human milk companies and human milk products in the absence of consistent governance and regulations raises concerns for vulnerable infants receiving these products. Thus, there is an urgent need to uncover and address the implications of the commercialization of human milk.

To theorize and define the commercialization of human milk, we conducted a concept analysis using Rodgers’ (1989) framework. The aim of this study was to utilize this framework to conceptualize the issue of the commercialization of human milk, develop a conceptual definition, and discuss the need for policy guidelines and regulations.

## Method

### Design

We systematically reviewed the literature to identify current evidence on equity, ethics, and regulations related to the commercialization, commodification, and marketing of, and profiting from, human milk products, human milk components, or laboratory-produced milk by for-profit companies. The rationale of the systematic review was to identify all current evidence related to the commercialization of human milk with the intention to make recommendations for child and maternal health policy development.

Rodger’s framework of concept analysis (Rodgers, 1989), which maintains that concepts are dynamic and continually evolving (Tofthagen & Fagerstrøm, 2010), was used to organize the systematic review. A systematic review protocol was not published. A concept analysis is a useful framework to provide clarification for novel or ambiguous concepts and develop conceptual definitions to aid in health practice and policy (Rodgers, 1989). This method was relevant for this study as it aided us in analyzing the evolutionary and novel topic of human milk commercialization, developing a conceptual definition, and understanding the antecedents and consequences of the industry. Rodger’s framework is comprised of six steps including concept identification; data collection; determination of antecedents, consequences and related concepts; and identification of a model case (Rodgers, 1989).

### Sample: Defining the Articles Reviewed

**Figure 1.**
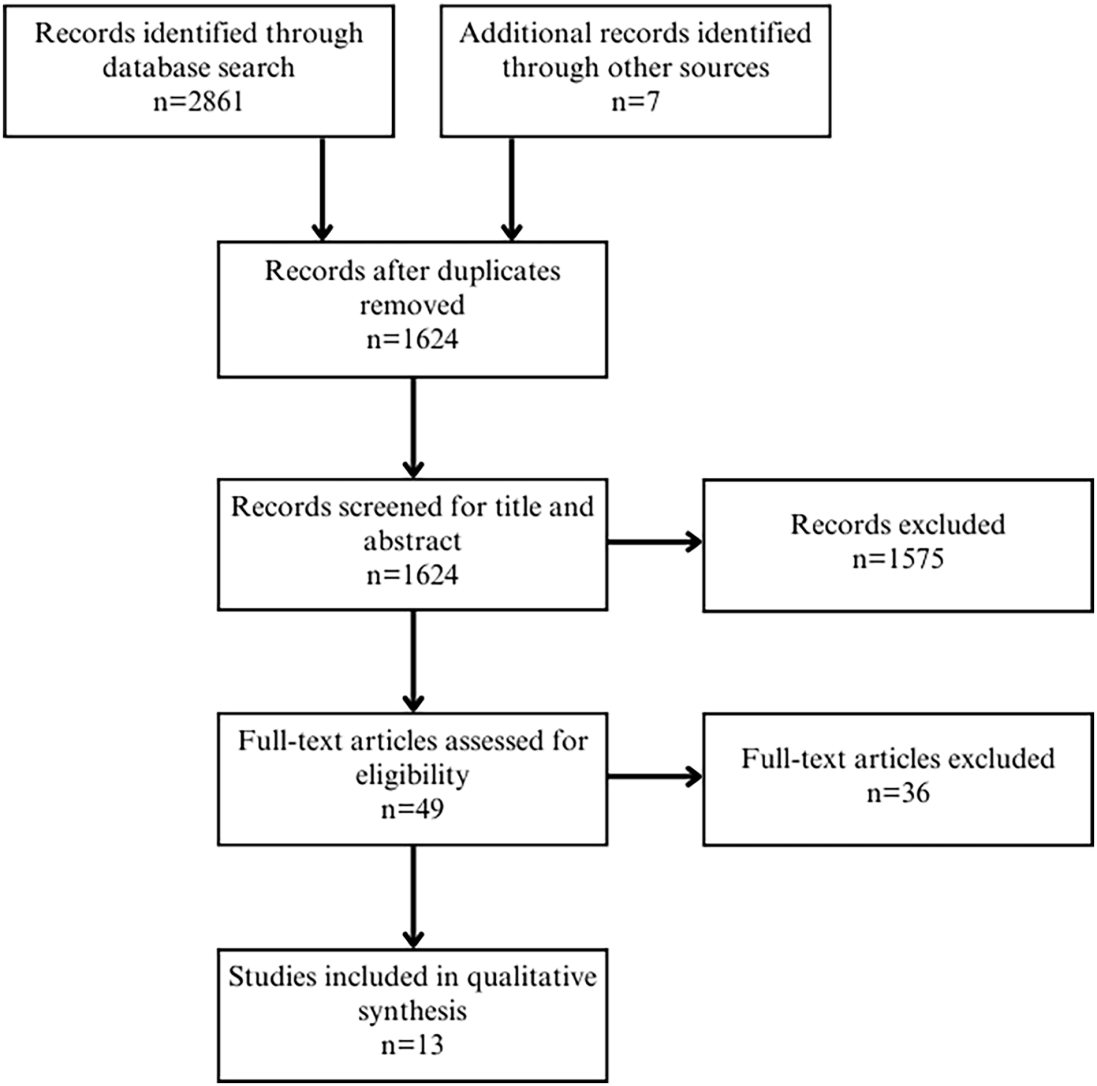
PRISMA Diagram.

Inclusion criteria included (1) English language, due to English being the common language of the authors; (2) peer-reviewed articles; and (3) published between the years 2000 and 2023, since the commercialization of human milk for infants appeared on the market in the 21^st^ century. Exclusion criteria included articles written in languages other than English. The study selection process was conducted independently by one project team member. The PRISMA diagram summarizing the literature screening process is outlined in Figure 1. The final sample size was 13 articles.

### Data Collection: The Search Strategy and Process

The dates of the inclusion period of the articles reviewed were from 2000 to 2023. The date last searched was June 2023 and included the following databases: Medline, Embase, CINAHL, Web of Science, Cochrane Database of Systematic Reviews, Alt Healthwatch, Law Journal Library, and ProQuest. Other sources include citations cited by the articles selected (Figure 1). The search terms included “human milk,” “breast milk,” “breastmilk,” “donor milk,” “maternal milk,” “mother’s milk,” “mother’s own milk,” and “commercialization,” “commodification,” “marketing,” “profit,” “ethics,” and “equity” (see the Appendix in the Online Supplemental Material).

### Measurement

The process used to extract data from articles was conducted by one author. Data were organized and summarized according to common themes using a matrix (Table 4). Themes included for-profit human milk, human milk companies, human milk products, lab-produced human milk, equity, ethics, regulations, and safety. Themes were determined by one author through inductive analysis, which is a fundamental component of Rodgers’ framework, and allows for the identification of commonalities through thematic analysis (Tofthagen & Fagerstrøm, 2010).

### Data Analysis

The methods used to describe characteristics of the sample include demographic information of published articles (Table 2), a summary of the articles reviewed, including study aim, sample, and design (Table 3), and a matrix of common themes (Table 4). Potential bias was evaluated by clearly defining the scope, objectives, and variables of the concept analysis. To limit bias, the literature review strategy was planned in collaboration with the senior author and an academic librarian. Reliability and validity were not considered because concept analyses aim to simply identify and examine commonalities amongst existing uses of a concept without employing rigorous standards (Rodgers, 1989).

**Table 2.** Demographic Information About Publishing Journals (*N* = 13).

Study’s First Author	Year of Publication	Journal	Article Country/Region of Origin
Cohen, M.	2019	*UC Irvine Law Review*	United States
Cohen, M.	2022	*Food and Drug Law Journal*	United States
Hartmann, B. T.	2019	*Seminars in Perinatology*	United States
Hassan, N.	2010	*Women’s Studies Quarterly*	United States
Israel-Ballard, K.	2019	*The Lancet Global Health*	International
Lee, R.	2019	*International Journal of Feminist Approaches to Bioethics*	Canada
Newman, S.	2022	*Review of International Political Economy*	United Kingdom
Paynter, M.	2018	*Healthcare Policy*	Canada
Prouse, C.	2021	*Environment and Planning A: Economy and Space*	Canada
Sigurdson, K. M. S.	2015	Dissertation	United States
Smith, J. P.	2015	*International Breastfeeding Journal*	Australia
Thibeau, S.	2018	*The Ochsner Journal*	United States
Waldeck, S. E.	2002	*Columbia Journal of Gender and Law*	United States

**Table 3.** Study Aims, Sample, and Design (*N* = 13).

Study’s First Author	Publication Date	Study Aim	Sample	Design	Citation
Cohen, M.	2019	Aimed to discuss if human milk should be regulated more tightly, and, if so, what types of legal reforms would be most desirable.		Expert opinion	Cohen, M. (2019). Should human milk be regulated? *UC Urvine Law Review*, *9*(3), 557–634.
Cohen, M.	2022	Hypothesized various outcomes of lab-produced human milk and examined their potential costs and benefits.		Expert opinion	Cohen, M. & Cassidy, T. (2022). Milk from mars. The challenges of regulating lab-produced (human) milk. *Food and Drug Law Journal*, 77(1), 6–46.
Hartmann, B. T.	2019	Aimed to determine the known benefits and risks of new human milk products.		Narrative Review	Hartmann, B. T. (2019). Benefit by design: Determining the “value” of donor human milk and medical products derived from human milk in NICU. *Seminars in Perinatology*, *43*(7), Article 151157. https://doi.org/10.1053/j.semperi.2019.06.005
Hassan, N.	2010	Focused on the emergence and growth of Prolacta Bioscience.		Expert opinion	Hassan, N. (2010). Milk markets: Technology, the lactating body, and new forms of consumption. *Women’s Studies Quarterly*, *38*(3–4), 209–228. https://doi.org/10.1353/wsq.2010.0016
Israel-Ballard, K.	2019	Aimed to discuss ethical considerations and key actions that should be considered as part of global and regional responses to donor milk policy and guideline development.		Report	Israel-Ballard, K., Cohen, J., Mansen, K., Parker, M., Engmann, C., Kelley, M., Brooks, E., Chatzixiros, E., Clark, D., Grummer-Strawn, L., Hartmann, B., Kennedy, S., Kent, G., Mwangome, M., Nyirenda, D., Perrin, M. T., Picaud, J., Reimers, P., Roest, J., & Romero-Maldonado, S. (2019). Call to action for equitable access to human milk for vulnerable infants. *The Lancet Global Health*, *7*(11), e1484–e1486. https://doi.org/10.1016/S2214-109X(19)30402-4.
Lee, R.	2019	Aimed to address the ethical implications of human milk exchange.		Expert Opinion	Lee, R. (2019). Commodifying compassion: Affective economies of human milk exchange. *International Journal of Feminist Approaches to Bioethics*, *12*(2), 92–116. https://doi.org/10.3138/ijfab.12.2.06.
Newman, S.	2022	Aimed to analyze social, political, and technical processes that transform breast milk into a commodity that is internationally traded and the implications of this for contemporary understandings of work and gender.	NeoLacta LifeSciences	Case study	Newman, S. & Nahman, M. (2022). Nurture commodified? An investigation into commercial human milk supply chains. *Review of International Political Economy*, *29*(6), 1967–1986. https://doi.org/10.1080/09692290.2020.1864757
Paynter, M.	2018	Aimed to describe the regulation of donor human milk in Canada, the lack of regulatory safeguards regarding for-profit operations and private milk exchange, and identified gaps putting families at risk.	Regulatory environment for donor human milk in Canada	Case Study	Paynter, M. J., & Hayward, K. (2018). Medicine, body fluid and food: The regulation of human donor milk in Canada. *Healthcare Policy*, *13*(3), 20–26. https://doi.org/10.12927/hcpol.2018.25400
Prouse, C.	2021	Aimed to explore three major ways in which human milk is being economically valued: calculating breastfeeding as a contribution to a country’s GDP (gross domestic product); buying and selling human milk to hospitals for profit; and manufacturing key components of human milk and the infant gut.		Expert opinion	Prouse, C. (2021). Mining liquid gold: The lively, contested terrain of human milk valuations. *Environment and Planning*, *53*(5), 958–976. https://doi.org/10.1177/0308518X21993817
Sigurdson, K. M. S.	2015	Aimed to outline two key issues that make contemporary forms of human milk exchange particularly contentious.		Qualitative doctoral dissertation	Sigurdson, K. M. S., Clarke, A. E. & Shim, J. K. (2015). *Emerging milk exchanges: Human milk banking, sharing and technoscience*. [Doctoral Dissertation, University of California, San Francisco]. ProQuest Dissertations Publishing. https://escholarship.org/uc/item/319639p1
Smith, J. P.	2015	Aimed to offer approaches to improve economic justice for women affected by the commodification and marketing of human milk and breastfeeding.		Commentary	Smith, J. P. (2015). Markets, breastfeeding and trade in mothers’ milk. *International Breastfeeding Journal*, *10*(1), Article 9. https://doi.org/10.1186/s13006-015-0034-9
Thibeau, S.	2018	Aimed to outline several ethical debates regarding donor human milk in the for-profit human milk industry surrounding respect for human dignity, beneficence, and justice for donor mothers and infants receiving donor milk.		Commentary	Thibeau, S., & Ginsberg. H. G. (2018). Bioethics in practice: The ethics surrounding the use of donor milk. *The Ochsner Journal*, *18*(1), 17–19.
Waldeck, S. E.	2002	Aimed to advocate for a market in human milk.		Expert opinion	Waldeck, S. E. (2002). Encouraging a market in human milk. *Columbia Journal of Gender and Law*, *11*(2), 361–406. https://doi.org/10.7916/cjgl.v11i2.2443

**Table 4.** Themes Related to “Commercialization of Human Milk” Present in the Literature (N = 13).

	Themes							
Author	For-Profit Human Milk	Human Milk Companies	Human Milk Products	Lab-Produced Human Milk	Equity	Ethics	Regulations	Safety
Cohen	•	•	•		•	•	•	•
Cohen & Cassidy	•	•	•	•		•	•	
Hartmann	•		•			•	•	•
Hassan	•	•	•	•				
Israel-Ballard et al.	•	•	•		•	•	•	
Lee	•	•	•			•	•	
Newman & Nahman	•	•	•	•			•	
Paynter & Hayward	•	•	•			•	•	
Prouse	•	•	•			•		
Sigurdson et al.	•	•	•	•		•		•
Smith	•	•	•				•	
Thibeau & Ginsberg	•	•	•		•	•	•	
Waldeck	•		•			•	•	

*Note.* Themes identified in articles indicated with a column notation “•”.

## Results

**Figure 2.**
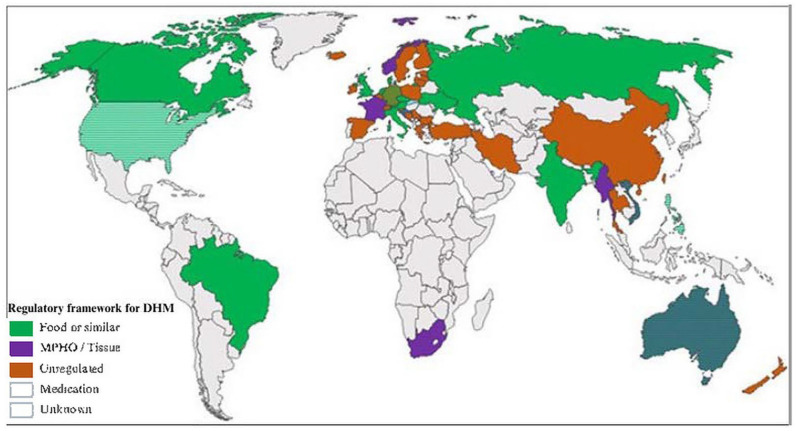
Regulatory Frameworks for Donor Human Milk (DHM). *Note.* Regulatory frameworks for donor human milk/human milk products globally. Striped indicates where two frameworks co-exist for different products (e.g., the United States and Australia), or where some regions of a country have regulations and others do not (e.g., Germany). MPHO = medical product of human origin. Reprinted with permission from “*Comparison between the for-profit human milk industry and non profit human milk banking: Time for regulations?*” by Shenker, N., Linden, J., Wang, B., Mackenzie, C., Hildebrandt, A. P., Spears, J., Davis, D., Nangia, S., and Weaver, G., 2024, *Maternal & Child Nutrition*, *20*(1), e13570 –e13570. Copyright 2023 by the Authors.

A total of 1,624 articles were reviewed for title and abstract. The full text of 49 articles was reviewed and 36 articles were excluded as they did not address for-profit companies and the commercialization of human milk. A total of 13 articles met the inclusion criteria (Figure 2). One non-peer-reviewed law review was included as it strongly addressed the commercialization of human milk and one PhD dissertation was included. The topics resulting from the systematic review are described; however, it is beyond the scope of this paper to discuss them fully.

### Definition and Attributes

Concept analyses are useful to develop definitions to reduce ambiguity of a concept and provide clarity (Rodgers, 1989). Reviewing the literature, we developed a definition for commercialized human milk (CHM). CHM can be defined as the packaging and sale of human milk and human milk components for financial gain. Human milk and human milk components are obtained through the expression of milk from a lactating person or are developed using biotechnology. People either donate or receive remuneration for their human milk or mammary cells.

### Antecedents: The Progression of Human Milk Commercialization

The commercialization of human milk can be attributed to several factors. These factors include increased demand for human milk, an unmet need for human milk, and technology. Table 5 summarizes the antecedents that have contributed to CHM.

**Table 5.** Antecedents of Commercialized Human Milk.

Antecedent	Main Points
Increased Demand	There has been a substantial rise in demand for DHM due to: the recent infant formula shortage; caregivers preferring to not formula feed; barriers to accessing DHM from nonprofit milk banks; temporary insufficient maternal milk supply of babies in NICU settings; and the superiority of human milk to infant formula.
Unmet Need	Globally, about 800,000 infants receive DHM on an annual basis. However, approximately 500,000 infants born less than 32 weeks do not have access to DHM (Shenker et al., 2021) leading to an unmet need for human milk.
Technology	Facilitates the storage and transport of human milk. Pasteurization technology has allowed for an extended shelf life of human milk and the elimination of bacteria, viruses, and other pathogens (Lima et al., 2017). Retort processing—an alternative technology—is now being used to create shelf-stable human milk products.
	Advancements in human milk science and scientific discovery provide companies with access to the rich lab-derived bio-fluid of human milk.

*Note.* DHM = donor human milk; NICU = neonatal intensive care unit.

### Increased Demand for Human Milk

The demand for human milk has increased substantially. Throughout 2019, the amount of pasteurized DHM dispensed to fragile babies throughout Canada and the United States increased from 6.4 million ounces to 7.4 million ounces (HMBANA, 2020). Further, in 2022, HMBANA milk banks provided nearly 10 million ounces of DHM, including to families during the formula shortage (HMBANA, 2023). This increase can be attributed to the preference of caregivers to not formula feed, increased accessibility of obtaining DHM from nonprofit milk banks (Palmquist & Doehler, 2016), and temporary insufficient maternal milk supply for babies in NICU settings. Further, the demand for human milk in the NICU setting is being driven by the compelling evidence that human milk is superior to infant formula, including its ability to reduce rates of NEC in preterm infants (Quigley et al., 2019). As a result, increased demand for human milk and evidence of important bioactive components found in human milk (Ballard & Morrow, 2013) have likely motivated for-profit human milk companies to invest in new technologies to produce CHM products.

### Unmet Need for Human Milk

The unmet need for human milk may also contribute to its commercialization. Globally, about 800,000 infants receive DHM annually. However, approximately 500,000 infants born at less than 32 weeks do not have access to DHM (Shenker et al., 2021) leading to an unmet need for human milk. This has likely contributed to the market for CHM products.

### Technology

Improvements in laboratory technology and advancements in human milk science have resulted in an increased availability of human milk (Miracle et al., 2011) and, most likely, CHM. Technology, including the breast pump, has facilitated the storage and transport of human milk (Lee, 2019) while pasteurization technology has allowed for an extended shelf life of human milk and the elimination of bacteria, viruses, and other pathogens (Lima et al., 2017). The Holder method of pasteurizing human milk at 62.58 °C for 30 minutes has historically been used as it reduces potential pathogenic contamination while also maintaining several bioactive components (Lima et al., 2017). Retort processing, a commercial food processing technology that sterilizes human milk using high heat of 121 °C (Jimenez et al., 2024) is now being used to create shelf-stable human milk products (Lima et al., 2017). Retort processing has been shown to destroy more bioactive components compared to Holder pasteurization, although only limited studies have been conducted (Meredith-Dennis et al., 2018). More research is needed to understand the results of retort processing on human milk and the consequences for the preterm population.

Advancements in human milk science and scientific discovery have also enabled the transformation of human milk into various products, and may also contribute to the commercialization of human milk. Human milk is increasingly viewed as a rich source of biomolecules that could serve other purposes, and scientific discovery provides companies with access to this rich bio-fluid. Human milk companies like Prolacta are even patenting their processes used in developing CHM products (McClain, 2018). Further, biotechnology is now being used to develop laboratory cultured human milk for infants from donated human mammary tissue (BIOMILQ, 2022; Wilk, n.d.-b).

### Consequences

There are several potential consequences resulting from the commercialization of human milk. These include undermining breastfeeding, quality and safety implications, inequitable distribution of products, exploitation and discrimination, potential displacement of maternal feeds, and extraction of human milk from communities. These consequences are outlined in Table 6.

**Table 6.** Consequences Resulting From the Commercialized Human Milk Industry.

Consequence	Summary
Undermining Breastfeeding	CHM products on the market may infer that MOM is insufficient or that it does not have enough nutrients (Hassan, 2010). For-profit human milk companies are free to promote and sell their products without adequate safeguards in place to protect those who are the most vulnerable. These practices are reminiscent of infant formula companies (Rollins et al., 2023) and can be misleading. For-profit human milk companies may work closely with healthcare providers and influence their clinical practices, which can also negatively affect breastfeeding.
Quality and Safety Implications	While non-profit milk banks in North America adhere to HMBANA guidelines, no consistent guidelines exist for the CHM industry (Shenker et al., 2024). Quality and safety of CHM products could be compromised due to lacking guidelines and regulations.
Inequitable Distribution	Access to CHM is often inequitable and can create ethical issues. Vulnerable infants most in need of human milk may be at risk of not receiving it (Thibeau & Ginsberg, 2018). Potential for inequitable distribution is higher if preterm infants at risk of NEC lack access to human milk (Thibeau & Ginsberg, 2018). CHM products can be costly and when available, they are often not covered by insurance, creating barriers to access (Thibeau & Ginsberg, 2018). While insurance coverage can improve equitable access, not all individuals have access to insurance.
Exploitation and Discrimination	The CHM industry has resulted in concerns of exploitation. Lactating individuals are often encouraged to become donors, receiving little to no renumeration, while their expressed milk is then processed and sold at a significant profit to hospitals (Newman & Nahman, 2022; Subramanian, 2022).
Displacement of Maternal Feeds	Maternal feeds may be displaced for the donor infant due to the donor expressing their milk to provide to for-profit human milk companies. If a donor is regularly expressing milk, their milk supply could increase. However, if irregular expression occurs, the milk supply for their own infant could be reduced (Hartmann, 2019).
Extraction of Human Milk From Communities	Human milk is being extracted from communities, particularly lower-income areas, and being sold at a profit. These practices can negatively influence human milk feeding in the communities from which the human milk is being extracted, potentially reducing the supply of human milk available for the children of that community.

*Note.* CHM = commercialized human milk; HMBANA = Human Milk Banking Association of North America; MOM = mother’s own milk; NEC = necrotizing enterocolitis.

### Undermining Breastfeeding

The existence of CHM products on the market may infer that MOM is insufficient or that it does not have enough nutrients (Hassan, 2010). Human milk companies are free to promote and sell their products without adequate safeguards in place to protect those who are the most vulnerable. These practices are reminiscent of infant formula companies (Rollins et al., 2023) and can be misleading. Human milk companies make claims, including that their products are “the best thing for babies” (Ni-Q, 2023), “breastmilk is becoming rarer and more difficult to obtain” (Wilk, n.d.-a, section 2: Our first steps towards a brighter future) and they are “transforming care for vulnerable babies by unlocking the power of human milk” (Prolacta BioScience, 2022a, page heading). Asserting that CHM products are equal to or superior to MOM is inaccurate and can undermine breastfeeding. Further, human milk companies may work closely with healthcare providers and influence their clinical practices, which can negatively affect breastfeeding. For example, in India, lactation support providers reportedly refer women with excess milk to NeoLacta, to whom they can donate their milk (Newman & Nahman, 2022).

### Quality and Safety Implications

Currently, the use of CHM in NICUs is governed by clinical decision-making by healthcare providers and insurance coverage rather than scientific evidence (Paynter & Hayward, 2018). Further, guidelines and regulations for the CHM industry are inconsistent (Shenker et al., 2024), which could lead to compromised quality and safety of CHM products (Hartmann, 2019; Klotz et al., 2022). For example, Neokare had to recall CHM products provided to preterm infants in 2023 for high lead levels (Dennys, 2023). Safety and quality of CHM products is paramount since these products are provided to the most vulnerable infants.

### Inequitable Distribution

Access to CHM is often inequitable and can create ethical issues. The potential for inequitable distribution is higher if preterm infants at risk of NEC lack access to human milk (Thibeau & Ginsberg, 2018). CHM products can be costly, and they are often not covered by insurance, creating barriers to access (Thibeau & Ginsberg, 2018). While insurance coverage can improve equitable access to human milk, not all individuals have insurance coverage, creating further inequities in distribution. Therefore, vulnerable infants most in need of human milk may be at risk of not receiving it (Thibeau & Ginsberg, 2018).

### Exploitation and Discrimination

The CHM industry has resulted in concerns of exploitation. Lactating individuals are encouraged to donate their milk, receiving little to no renumeration, while their expressed milk is sold at a significant profit (Newman & Nahman, 2022; Subramanian, 2022). Reports have shown that NeoLacta donors typically have low education and receive small amounts of money or food for their expressed milk (Newman & Nahman, 2022). Concerns of discrimination have also arisen. Medolac Laboratories faced criticism for campaigning for donations of expressed milk from Black women in a low-income area in Detroit with lower breastfeeding rates (Lee, 2019).

In contrast, some companies rely on the presumption that exploitation is negated when monetary compensation is not involved (Newman and Nahman, 2022). NeoLacta convinces lactating individuals that their “excess” milk is waste and is an issue that can be resolved by donating their over-supply (Newman & Nahman, 2022). Prolacta has been criticized for not being transparent regarding the use of their human milk donations. While encouraging individuals to donate their expressed milk for infants in South Africa, Prolacta sells their CHM products at a profit in the United States (Lee, 2019).

### Displacement of Maternal Feeds

Maternal feeds may be displaced for the donor infant due to the donor expressing their milk to provide to human milk companies. If a donor is regularly expressing milk, their milk supply could increase. However, if irregular expression occurs, the milk supply for their own infant could be reduced (Hartmann, 2019). As with breast milk substitutes, there is a risk of displacement of maternal feeds. Appropriate support and counseling need to be provided to ensure minimal disruption of lactation.

### Extraction of Human Milk From Communities

Human milk is extracted from communities, particularly in lower-income countries, and sold at a profit to higher income countries. Ambrosia Labs paid impoverished women in Cambodia to express their milk for export to the United States. The Government of Cambodia outlawed the practice in 2017 with concerns that exporting human milk would exploit Cambodian women and could affect the supply of human milk available for the country’s own children (Newman & Nahman, 2022). NeoLacta sourced human milk from low-income women in India and exported their CHM products to Australia (Newman & Nahman, 2022). These practices can negatively influence human milk feeding in the communities from which the human milk is being extracted, potentially reducing the supply of human milk available for the children of that community.

### Governance and Policy

The identified literature revealed that, despite the growth in CHM products and companies, guidelines and regulations are lacking and inconsistent (Klotz et al., 2022; Paynter & Hayward, 2018, Shenker et al. 2024). This raises concerns for ethics, safety, and the equitable distribution of human milk products (Cohen, 2019; Hartmann, 2019; Shenker et al., 2024). On a global scale, there are no international guidelines regulating the marketing and sale of human milk. The International Code of Marketing of Breast-milk Substitutes (the Code) provides recommendations on regulation of the promotion of infant formula, bottles, and teats (WHO, 2017). However, human milk is not covered by the Code (WHO, 2017).

There is also debate regarding how human milk should be categorized, leading to inconsistencies in the regulation of human milk. Current categorizations of human milk include human milk as a food, medicine, medical product of human origin, or tissue (Shenker et al., 2024). Existing regulatory frameworks for DHM globally are outlined in Figure 2.

### Concepts Related to the Commercialization of Human Milk

Cell-cultured human milk is not the first type of food to be produced in a laboratory (Cohen & Cassidy, 2022). Cell culturing has also been used to develop food products including milk (Wilk, n.d.-b), yogurt (Wilk, 2022), and meat (Good Food Institute, 2023). The cultivated meat industry is changing quickly, and several countries are establishing policies for the sale of laboratory grown meat (Good Food Institute, 2023). Human milk components, including human milk oligosaccharides and lactoferrin, are an additional growing area of research and product development. Companies are utilizing various technologies to further develop infant formula by adding these important human milk components (dsm-firmenich, 2023; Helaina, 2023; Turtle Tree, 2024). However, these growing industries all have a need for consistent governance and policy.

### Model Case

Baby Gabriel was born prematurely at 29 weeks’ gestation, weighing 1600 grams. Due to being premature, specialized care was needed in the NICU to support Gabriel’s growth and development. Unfortunately, Gabriel’s mother, Clara, experienced postpartum hemorrhage and as a result her milk production was delayed. Clara was advised by the neonatologist that Gabriel needed supplementation, with human milk being the preferred option. Since Gabriel was not considered low-birth weight, they did not qualify for DHM according to the hospital’s NICU policy. The hospital offered Clara the choice to provide commercialized donor human milk to Gabriel; however, it was costly and was not covered by her health insurance. Clara’s other option was to provide formula to Gabriel which placed Gabriel at a significantly increased risk for developing NEC. Clara struggled with the decision to pay for a CHM product that may potentially improve Gabriel’s outcomes.

## Discussion

The literature identified provides an understanding of the implications of inconsistent guidance and regulatory frameworks relative to the CHM industry. The rapidly growing CHM industry will likely continue to accelerate and present further challenges. The marketing tactics resembling those used by formula companies are of international relevance as CHM could be incorporated into The Code to regulate marketing used by human milk companies.

The current compensation structure for lactating individuals who provide their milk to for-profit companies is not readily available. It has been reported that companies vary in their compensation methods, ranging from voluntary donations to remunerating with food and small amounts of money. There may be incentive for lactating individuals to express their milk for remuneration, which can potentially lead to oversupply for that individual. Exploring the impact of remuneration on maternal health outcomes would be beneficial. Further, if payment for milk is based on volume, there may be incentive for lactating individuals to dilute their milk with the intention to increase the volume, and therefore receive additional compensation. While some companies test for foreign milk proteins and DNA (Prolacta BioScience, 2024), it is not a mandatory practice, therefore, the potential for diluted milk exists. More research is needed in this area to determine how compensation may affect human milk donation.

The literature reviewed did not strongly discuss the benefits of CHM products; however, there is potential to increase the supply of human milk and specialized human milk products as well as to advance human milk research. While marketing of CHM could be an issue if it were to dissuade mothers from breastfeeding, it could be beneficial if it encourages NICUs to use human milk rather than infant formula. The positive aspects of CHM products could benefit from further research.

This subject is clinically relevant for lactation support providers as well as other healthcare providers. Increased awareness of CHM in the clinical practice setting will help lactation support providers to employ critical thinking in their practice around the use of these products. It will also help providers to educate families so that they are able to engage in better informed decision-making around infant feeding.

### Limitations

Due to the novelty of this subject, the literature review resulted in a limited number of studies, and there was a paucity of evidence on the prevalence of CHM products. This limitation in methodology can create challenges for understanding the full extent of the issues surrounding CHM. The use of English-only manuscripts in this concept analysis can also be considered a limitation. The limited literature presents a need for further research and may also constrain implementation of the research into practice and policy.

## Conclusion

Commercialized human milk can be defined as the packaging and sale of human milk and human milk components for financial gain. The commercialization of human milk is a rapidly growing industry with an urgent need for stronger national and global policies and regulations to ensure accessibility, ethics, and safety. If left unaddressed, there could be potential risks for donor milk banking, the future of breastfeeding, and infant and maternal health.

## Supplemental Material

sj-docx-1-jhl-10.1177_08903344241254345 – Supplemental material for Conceptualizing the Commercialization of Human Milk: A Concept Analysis Supplemental material, sj-docx-1-jhl-10.1177_08903344241254345 for Conceptualizing the Commercialization of Human Milk: A Concept Analysis by Heather Christine Rusi, Laurence Grummer-Strawn, Maryanne Tigchelaar Perrin, Tracie Risling and Meredith Lee Brockway in Journal of Human Lactation

sj-jpg-2-jhl-10.1177_08903344241254345 – Supplemental material for Conceptualizing the Commercialization of Human Milk: A Concept Analysis Supplemental material, sj-jpg-2-jhl-10.1177_08903344241254345 for Conceptualizing the Commercialization of Human Milk: A Concept Analysis by Heather Christine Rusi, Laurence Grummer-Strawn, Maryanne Tigchelaar Perrin, Tracie Risling and Meredith Lee Brockway in Journal of Human Lactation

## References

[bibr1-08903344241254345] Ames S. R. Lotoski L. C. Azad M. B. (2023). Comparing early life nutritional sources and human milk feeding practices: Personalized and dynamic nutrition supports infant gut microbiome development and immune system maturation. Gut Microbes, 15(1), Article 2190305. 10.1080/19490976.2023.2190305 PMC10114993 37055920

[bibr2-08903344241254345] Ballard O. Morrow A. L. (2013). Human milk composition: Nutrients and bioactive factors. The Pediatric Clinics of North America, 60(1), 49–74. 10.1016/j.pcl.2012.10.002 23178060 PMC3586783

[bibr3-08903344241254345] BIOMILQ. (2022). About us: Cell-cultured human milk. https://www.biomilq.com

[bibr4-08903344241254345] Cohen M. (2019). Should human milk be regulated. UC Irvine Law Review, 9(3), 557–634.

[bibr5-08903344241254345] Cohen M. Cassidy T. (2022). Milk from mars: The challenges of regulating lab-produced (human) milk. Food and Drug Law Journal, 77(1), 6–50.

[bibr6-08903344241254345] Dennys H. (2023, January 8). Families on alert after an investigation finds lead in breast milk products supplied to newborns. DailyMail. https://www.dailymail.co.uk/news/article-11610879/Families-alert-investigation-finds-lead-breast-milk-products-supplied-newborns.html

[bibr7-08903344241254345] dsm-firmenich. (2023). Human milk oligosaccharides. DSM. https://www.dsm.com/human-nutrition/en/products/hmos.html

[bibr8-08903344241254345] Good Food Institute. (2023). What is cultivated meat? https://gfi.org/cultivated/?gclid=CjwKCAjw_aemBhBLEiwAT98FMuI4tmHD13ex1mjrBMQylQOAJQQODphr7wzB_bwpHPR3u-a5d-gF2RoCbNgQAvD_BwE

[bibr9-08903344241254345] Hartmann B. T. (2019). Benefit by design: Determining the ‘value’ of donor human milk and medical products derived from human milk in NICU. Seminars in Perinatology, 43(7), 151157 –151157. 10.1053/j.semperi.2019.06.005 31383367

[bibr10-08903344241254345] Hassan N. (2010). Milk markets: Technology, the lactating body, and new forms of consumption. Women’s Studies Quarterly, 38(3–4), 209–228. 10.1353/wsq.2010.0016

[bibr11-08903344241254345] Helaina. (2023). Helaina is the future of nutrition. https://www.myhelaina.com/

[bibr12-08903344241254345] Human Milk Banking Association of North America. (2020, March 31). Donor human milk distribution increases by nearly 1 million ounces. https://www.hmbana.org/news/donor-human-milk-increases-by-nearly-1-million-ounces.html

[bibr13-08903344241254345] Human Milk Banking Association of North America. (2023, February 20). Nonprofit milk banks step up during formula crisis, dispensing nearly 10 million ounces in 2022. https://www.hmbana.org/news/blog.html/article/2023/02/20/nonprofit-milk-banks-step-up-during-formula-crisis-dispensing-nearly-10-million-ounces-in-2022

[bibr14-08903344241254345] Israel-Ballard K. (2018). Strengthening systems to ensure all infants receive human milk: Integrating human milk banking into newborn care and nutrition programming. Breastfeeding Medicine, 13(8), 524–526. 10.1089/bfm.2018.0133 30335484

[bibr15-08903344241254345] Israel-Ballard K. Cohen J. Mansen K. Parker M. Engmann C. Kelley M. , & for the Oxford-PATH Milk Working Group. (2019). Call to action for equitable access to human milk for vulnerable infants. The Lancet, Global Health, 7(11), e1484–e1486. https://doi.org/10.1016/S2214-109X(19)30402-4 10.1016/S2214-109X(19)30402-4 PMC7613495 31607455

[bibr16-08903344241254345] Jimenez P. S. Bangar S. P. Suffern M. Whiteside W. S. (2024). Understanding retort processing: A review. Food Science & Nutrition, 12(3), 1545–1563. 10.1002/fsn3.3912 38455166 PMC10916645

[bibr17-08903344241254345] Jones F. (2003). History of north american donor milk banking: One hundred years of progress. Journal of Human Lactation, 19(3), 313–318. 10.1177/0890334403255857 12931784

[bibr18-08903344241254345] Kenton W. (2023, December 7). Not for profit: Definitions and what it means for taxes. Investopedia. https://www.investopedia.com/terms/n/not-for-profit.asp#toc-for-profit-vs-not-for-profit

[bibr19-08903344241254345] Klotz D. Wesolowska A. Bertino E. Moro G. E. Picaud J. C. Gaya A. Weaver G. (2022). The legislative framework of donor human milk and human milk banking in Europe. Maternal & Child Nutrition, 18(2), Article e13310. 10.1111/mcn.13310 PMC8932705 34936203

[bibr20-08903344241254345] Lee R. (2019). Commodifying compassion: Affective economies of human milk exchange. International Journal of Feminist Approaches to Bioethics, 12(2), 92–116. 10.3138/ijfab.12.2.06

[bibr21-08903344241254345] Lima H. K. Wagner-Gillespie M. Perrin M. T. Fogleman A. D. (2017). Bacteria and bioactivity in holder pasteurized and shelf-stable human milk products. Current Developments in Nutrition, 1(8), Article e001438. 10.3945/cdn.117.001438 PMC5998364 29955718

[bibr22-08903344241254345] McClain V. W. (2018). Patents on life: A brief view of human milk component patenting. World Nutrition, 9(1), 57. 10.26596/wn.20189157-69

[bibr23-08903344241254345] Meredith-Dennis L. Xu G. Goonatilleke E. Lebrilla C. B. Underwood M. A. Smilowitz J. T. (2018). Composition and variation of macronutrients, immune proteins, and human milk oligosaccharides in human milk from nonprofit and commercial milk banks. Journal of Human Lactation, 34(1), 120–129. 10.1177/0890334417710635 28614672

[bibr24-08903344241254345] Miracle D. J. Szucs K. A. Torke A. M. Helft P. R. (2011). Contemporary ethical issues in human milk-banking in the United States. Pediatrics, 128(6), 1186–1191. 10.1542/peds.2010-2040 22084324

[bibr25-08903344241254345] NeoKare. (2023). Pasteurised human milk products. https://neokare.co.uk/pasteurized-human-milk-products/

[bibr26-08903344241254345] NeoLacta Lifesciences. (2022). Welcome to Neolacta. https://neolacta.com/

[bibr27-08903344241254345] Newman S. Nahman M. (2022). Nurture commodified? An investigation into commercial human milk supply chains. Review of International Political Economy, 29(6), 1967–1986. 10.1080/09692290.2020.1864757

[bibr28-08903344241254345] Ni-Q. (2023). Home. https://www.ni-q.com/

[bibr29-08903344241254345] Palmquist A. E. L. Doehler K. (2016). Human milk sharing practices in the U.S. Maternal and Child Nutrition, 12(2), 278–290. 10.1111/mcn.12221 26607304 PMC5063162

[bibr30-08903344241254345] Parker M. G. Burnham L. A. Kerr S. Belfort M. B. Perrin M. Corwin M. Heeren T. (2020). Prevalence and predictors of donor milk programs among U.S. advanced neonatal care facilities. Journal of Perinatology, 40(4), 672–680. 10.1038/s41372-020-0620-6 32103161

[bibr31-08903344241254345] Paynter M. J. Hayward K. (2018). Medicine, body fluid and food: The regulation of human donor milk in Canada. Healthcare Policy, 13(3), 20–26. 10.12927/hcpol.2018.25400 29595434 PMC5863867

[bibr32-08903344241254345] Prolacta BioScience. (2022a). About us. https://www.prolacta.com/en/about-us/

[bibr33-08903344241254345] Prolacta BioScience. (2022b). Preterm nutrition products. https://www.prolacta.com/en/products/preterm-nutrition-products/

[bibr34-08903344241254345] Prolacta BioScience. (2024). Testing, screening, and standardized production process. https://www.prolacta.com/en/resource-library/testing-screening-and-standardized-production-process/

[bibr35-08903344241254345] Prouse C. (2021). Mining liquid gold: The lively, contested terrain of human milk valuations. Environment and Planning, 53(5), 958–976. https://doi.org/10.1177/0308518X21993817 10.1177/0308518X21993817 PMC8317231 34381290

[bibr36-08903344241254345] Quigley M. Embleton N. D. McGuire W. (2019). Formula versus donor breast milk for feeding preterm or low birth weight infants. Cochrane Database of Systematic Reviews, 2019(8), Article CD002971. 10.1002/14651858.CD002971.pub5 PMC6640412 31322731

[bibr37-08903344241254345] Rodgers B. L. (1989). Concepts, analysis and the development of nursing knowledge: The evolutionary cycle. Journal of Advanced Nursing, 14(4), 330–335. 10.1111/j.1365-2648.1989.tb03420.x 2661622

[bibr38-08903344241254345] Rollins N. Piwoz E. Baker P. Kingston G. Mabaso K. M. McCoy D. Ribeiro Neves P. A. Pérez-Escamilla R. Richter L. Russ K. Sen G. Tomori C. Victora C. G. Zambrano P. Hastings G. (2023). Marketing of commercial milk formula: A system to capture parents, communities, science, and policy. The Lancet (British Edition), 401(10375), 486–502. 10.1016/S0140-6736(22)01931-6 36764314

[bibr39-08903344241254345] Shenker N. Linden J. Wang B. Mackenzie C. Hildebrandt A. P. Spears J. Davis D. Nangia S. Weaver G. (2024). Comparison between the for-profit human milk industry and nonprofit human milk banking: Time for regulation? Maternal & Child Nutrition, 20(1), Article e13570. 10.1111/mcn.13570 PMC10749996 37830377

[bibr40-08903344241254345] Shenker N. Staff M. Vickers A. Aprigio J. Tiwari S. Nangia S. Sachdeva R. C. Clifford V. Coutsoudis A. Reimers P. Israel-Ballard K. Mansen K. Mileusnic-Milenovic R. Wesolwska A. Goudoever J. B. V. Hosseini M. Klotz D. Grøvslien A. H. Weaver G. (2021). Maintaining human milk bank services throughout the COVID-19 pandemic: A global response. Maternal and Child Nutrition, 17(3), Article e13131 10.1111/mcn.13131 PMC7883204 33403779

[bibr41-08903344241254345] Sigurdson K. M. S. Clarke A. E. Shim J. K. (2015). Emerging milk exchanges: Human milk banking, sharing and technoscience[Doctoral Dissertation, University of California, San Francisco]. ProQuest Dissertations Publishing. https://escholarship.org/uc/item/319639p1

[bibr42-08903344241254345] Smith J. P. (2015). Markets, breastfeeding and trade in mothers’ milk. International Breastfeeding Journal, 10(1), Article 9. 10.1186/s13006-015-0034-9 PMC4380115 25829943

[bibr43-08903344241254345] Stevens E. E. Patrick T. E. Pickler R. (2009). A history of infant feeding. The Journal of Perinatal Education, 18(2), 32–39. 10.1624/105812409X426314 PMC2684040 20190854

[bibr44-08903344241254345] Stolzer J. (2018). Breastfeeding: An interdisciplinary review. International Review of Modern Sociology, 44(1–2), 101–126.

[bibr45-08903344241254345] Subramanian S. (2022, May 13). She pioneered the sale of breastmilk, then lost everything. The Washington Post. https://www.washingtonpost.com/magazine/2022/05/13/price-of-selling-breast-milk/

[bibr46-08903344241254345] Thibeau S. Ginsberg H. G. (2018). Bioethics in practice: The ethics surrounding the use of donor milk. The Ochsner Journal, 18(1), 17–19. 29559863 PMC5855414

[bibr47-08903344241254345] Tofthagen R. Fagerstrøm L. M. (2010). Rodgers’ evolutionary concept analysis: A valid method for developing knowledge in nursing science. Scandinavian Journal of Caring Sciences, 24(s1), 21–31. 10.1111/j.1471-6712.2010.00845.x 21070310

[bibr48-08903344241254345] Turtle Tree . (2024). Unlocking the future of nutrition with LF+, Turtle Tree’s unique lactoferrin. https://www.turtletree.com/unlocking-the-future-of-nutrition-with-lf-turtletrees-unique-lactoferrin/

[bibr49-08903344241254345] United Nations Children’s Fund. (2018). Breastfeeding: A mother’s gift, for every child. https://data.unicef.org/resources/breastfeeding-a-mothers-gift-for-every-child/

[bibr50-08903344241254345] Victora C. G. Bahl R. Barros A. J. D. França G. V. A. Horton S. Krasevec J. Murch S. Sankar M. J. Walker N. Rollins N. C. (2016). Breastfeeding in the 21st century: Epidemiology, mechanisms, and lifelong effect. The Lancet (British Edition), 387(10017), 475–490. 10.1016/S0140-6736(15)01024-7 26869575

[bibr51-08903344241254345] Waldeck S. E. (2002). Encouraging a market in human milk. Columbia Journal of Gender and Law, 11(2), 361–406. https://doi.org/10.7916/cjgl.v11i2.2443

[bibr52-08903344241254345] Wilk . (n.d.-a). Welcome home. https://wilkismilk.com/

[bibr53-08903344241254345] Wilk . (n.d.-b). Yes we can. https://wilkismilk.com/

[bibr54-08903344241254345] Wilk . (2022, December 8). Wilk presents world’s first cell-based yogurt produced with cultured milk fat. https://wilkismilk.com/news/wilk-presents-worlds-first-cell-based-yogurt-produced-with-cultured-milk-fat/

[bibr55-08903344241254345] World Health Organization. (1991). Indicators for assessing breastfeeding practices. https://www.who.int/publications/i/item/WHO_CDD_SER_91.14_Corr.1

[bibr56-08903344241254345] World Health Organization. (2001). The optimal duration of exclusive breastfeeding: Report of an expert consultation, Geneva, Switzerland. https://www.who.int/publications/i/item/WHO-NHD-01.09

[bibr57-08903344241254345] World Health Organization. (2011). Guidelines on optimal feeding of low birth-weight infants in low- and middle-income countries. https://www.who.int/publications/i/item/9789241548366 26042325

[bibr58-08903344241254345] World Health Organization. ( 2017). The international code of marketing of breast-milk substitutes: Frequently asked questions (2017 Update). https://www.who.int/publications/i/item/WHO-NMH-NHD-17.1

[bibr59-08903344241254345] World Health Organization, and United Nations Children’s Fund. (2018). Implementation guidance: Protecting, promoting, and supporting breastfeeding in facilities providing maternity and newborn services—The revised Baby-friendly Hospital Initiative. https://www.who.int/publications/i/item/9789241513807

[bibr60-08903344241254345] World Health Organization, and United Nations Children’s Fund. (2022). Global breastfeeding scorecard 2022: Protecting breastfeeding through further investments and policy actions. https://www.who.int/publications/i/item/WHO-HEP-NFS-22.6

