# Supplementary material for: Conceptualizing the Commercialization of Human Milk: A Concept Analysis
Source: J Hum Lact. 2024 Jun 10;40(3):392–404. doi: 10.1177/08903344241254345 (PMC11340243; doi:10.1177/08903344241254345)
Supplement: sj-docx-1-jhl-10.1177_08903344241254345 – Supplemental material for Conceptualizing the Commercialization of Human Milk: A Concept Analysis [file sj-docx-1-jhl-10.1177_08903344241254345.docx]

**Appendix**

Full Electronic Search Strategy

| Database Searched | Search Strategy |
| --- | --- |
| Medline Ovid | 1. ‘human milk’.tw 2. ‘breast milk’.tw 3. Breastmilk.tw 4. ‘donor milk’.tw 5. ‘maternal milk’.tw 6. ‘mother’s milk’.tw 7. ‘mother’s own milk’.tw 8. commerciali*.tw 9. commod*.tw 10. market*.tw 11. ethic*.tw 12. profit*.tw 13. equit*.tw 14. Milk, Human/ 15. Ethics/ 16. Commerce/ or commodification/ or marketing/ 17. 1 or 2 or 3 or 4 or 5 or 6 or 7 or 14 18. 8 or 9 or 10 or 11 or 12 or 13 or 15 or 16 19. 17 and 18 20. Limit 19 to English language |
| Medline Embase | 1. ('human milk' or 'breast milk' or breastmilk or 'donor milk' or 'maternal milk' 2. or 'mother's milk' or 'mother's own milk').ab,ti. 3. Breast milk/ or donor milk/ 4. 1 or 2 5. Commodification/ 6. commerciali*.ab,ti. 7. commod*.ab,ti. 8. ethic*.ab,ti. 9. Ethics/ 10. market*.ab,ti. 11. Marketing/ 12. profit*.ab,ti. 13. Profit/ 14. equit*.ab,ti. 15. 4 or 5 or 6 or 7 or 8 or 9 or 10 or 11 or 12 or 13 16. 3 and 14 17. Limit 15 to English language |
| CINAHL | S20 = S9 AND S19  S9 AND S19  S9 = S1 OR S2 OR S3 OR S4 OR S5 OR S6 OR S7 OR S8  S19 = S10 OR S11 OR S12 OR S13 OR S14 OR S15 OR S16 OR S17 OR S18  S1 = (MH “Milk, Human” OR MH “Donor Milk”)  S2 = TI human milk OR AB human milk  S3 = TI breast milk OR AB breast milk  S4 = TI breastmilk OR AB breastmilk  S5 = TI donor milk OR AB donor milk  S6 = TI maternal milk OR AB maternal milk  S7 = TI mothers milk OR AB mothers milk  S8 = TI mothers own milk OR AB mothers own milk  S10 = (MH “Marketing”)  S11 = (MH “Profits”)  S12 = (MH “Ethics”)  S13 = TI commerciali* OR AB commerciali*  S14 = TI commod* OR AB commod*  S15 = TI market* OR AB market*  S16 = TI profit* OR AB profit*  S17 = TI ethic* OR AB ethic*  S18 = TI equit* OR AB equit* |
| Web of Science | "human milk" or "breast milk" or breastmilk or "donor milk" or "maternal milk"  or "mothers milk" or "mother's own milk" (Title)  or  "human milk" or "breast milk" or breastmilk or "donor milk" or "maternal milk"  or "mothers milk" or "mother's own milk" (Abstract)  and  commerciali* or commod* or market* or ethic* or profit* or equit* (Title)  or  commerciali* or commod* or market* or ethic* or profit* or equit* (Abstract) |
| Cochrane Database of Systematic Reviews | ""human milk" OR "breast milk" OR breastmilk OR "donor milk" OR "maternal milk" OR "mothers milk" OR "mother's own milk"" in Title  or  ""human milk" OR "breast milk" OR breastmilk OR "donor milk" OR "maternal milk" OR "mothers milk" OR "mother's own milk"" in Abstract  and   "commerciali* OR commod* OR ethic* OR market* OR profit* OR equit*" in Title  or  "commerciali* OR commod* OR ethic* OR market* OR profit* OR equit*" in Abstract |
| Alt Health Watch | S20 = S9 AND S19  S9 = S1 OR S2 OR S3 OR S4 OR S5 OR S6 OR S7 OR S8  S19 = S10 OR S11 OR S12 OR S13 OR S14 OR S15 OR S16 OR S17 OR S18  S1 = (MH “Milk, Human” OR MH “Donor Milk”)  S2 = TI human milk OR AB human milk  S3 = TI breast milk OR AB breast milk  S4 = TI breastmilk OR AB breastmilk  S5 = TI donor milk OR AB donor milk  S6 = TI maternal milk OR AB maternal milk  S7 = TI mothers milk OR AB mothers milk  S8 = TI mothers own milk OR AB mothers own milk  S10 = (MH “Marketing”)  S11 = (MH “Profits”)  S12 = (MH “Ethics”)  S13 = TI commerciali* OR AB commerciali*  S14 = TI commod* OR AB commod*  S15 = TI market* OR AB market*  S16 = TI profit* OR AB profit*  S17 = TI ethic* OR AB ethic*  S18 = TI equit* OR AB equit* |
| Law Journal Library | (((“human milk” OR “breast milk” OR breastmilk OR “donor milk” OR “maternal milk”  OR “mothers milk” OR “mother’s own milk”) AND (commerciali* OR commod*  OR market* OR profit* OR ethic* OR equit* OR regulat*))) |
| ProQuest Dissertations and Theses Global | abstract("human milk" OR "breast milk" OR breastmilk OR "donor milk"  OR "maternal milk" OR "mothers milk" OR "mother's own milk")  AND abstract(commerciali* OR commod* OR ethic* OR equit* OR profit* OR market) |

*Note.* The *regulation* search term was only used for the Law Journal Library as it resulted in a considerable number of irrelevant results in other databases.
