# Supplementary figures and images for: Conceptualizing the Commercialization of Human Milk: A Concept Analysis
Source: J Hum Lact. 2024 Jun 10;40(3):392–404. doi: 10.1177/08903344241254345 (PMC11340243; doi:10.1177/08903344241254345)

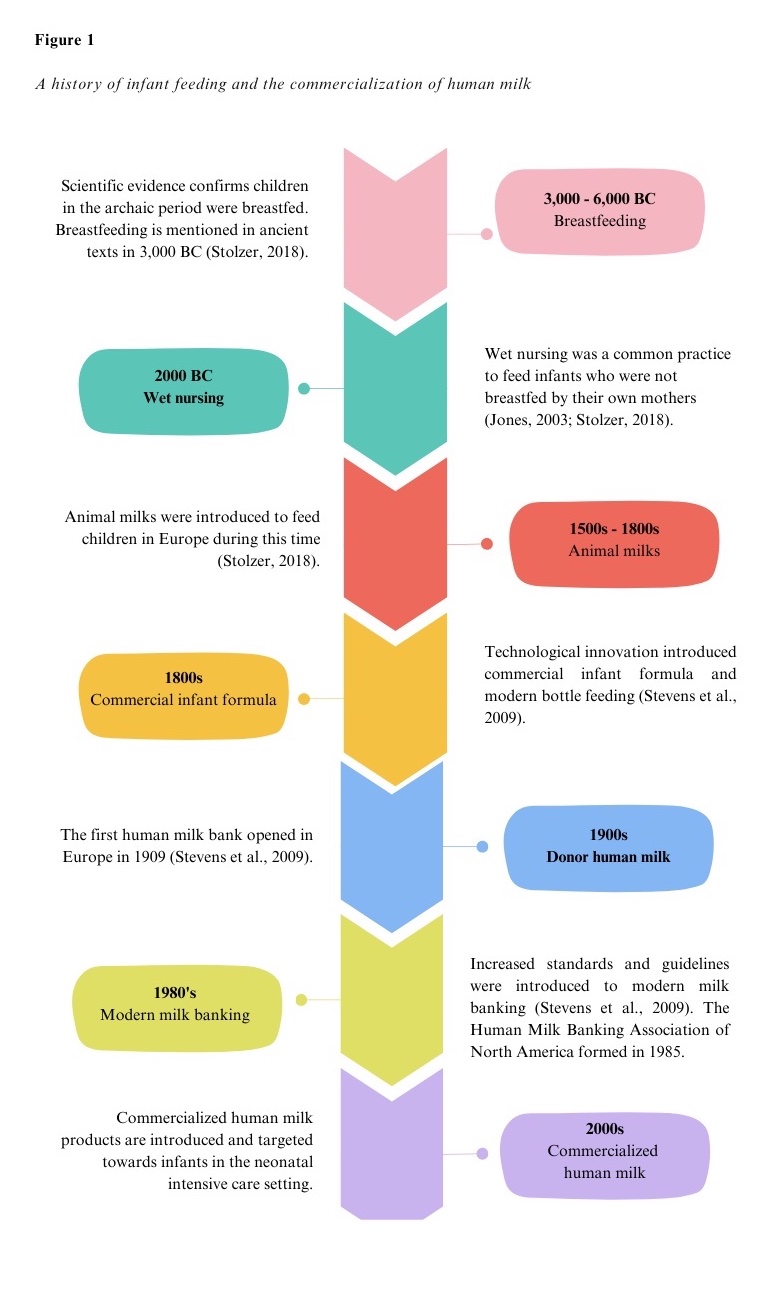

Supplement: sj-jpg-2-jhl-10.1177_08903344241254345 – Supplemental material for Conceptualizing the Commercialization of Human Milk: A Concept Analysis [file sj-jpg-2-jhl-10.1177_08903344241254345.jpg]
